# Implementation of single-breath-hold cone beam CT guided hypofraction radiotherapy for lung cancer

**DOI:** 10.1186/1748-717X-9-77

**Published:** 2014-03-20

**Authors:** Renming Zhong, Jin Wang, Lin Zhou, Feng Xu, Li Liu, Jidan Zhou, Xiaoqin Jiang, Nianyong Chen, Sen Bai, You Lu

**Affiliations:** 1 Department of Radiation Oncology, Cancer Center, West China School of Medicine/West China Hospital, Sichuan University, 37 Guoxue Lane, Chengdu 610041, People’s Republic of China; 2 Department of Thoracic Oncology, Cancer Center and State Key Laboratory of Biotherapy, West China School of Medicine/West China Hospital, Sichuan University, 37 Guoxue Lane, Chengdu 610041, People’s Republic of China

**Keywords:** Active breathing control, Tumor position reproducibility, Rationale, PTV margin

## Abstract

**Background:**

To analyze the feasibility of active breath control (ABC), the lung tumor reproducibility and the rationale for single-breath-hold cone beam CT (CBCT)-guided hypofraction radiotherapy.

**Methods:**

Single-breath-hold CBCT images were acquired using ABC in a cohort of 83 lung cancer patients (95 tumors) treated with hypofraction radiotherapy. For all alignments between the reference CT and CBCT images (including the pre-correction, post-correction and post-treatment CBCT images), the tumor reproducibility was evaluated via online manual alignment of the tumors, and the vertebral bone uncertainties were evaluated via offline manual alignment of the vertebral bones. The difference between the tumor reproducibility and the vertebral bone uncertainty represents the change in the tumor position relative to the vertebral bone. The relative tumor positions along the coronal, sagittal and transverse axes were measured based on the reference CT image. The correlations between the vertebral bone uncertainty, the relative tumor position, the total treatment time and the tumor reproducibility were evaluated using the Pearson correlations.

**Results:**

Pre-correction, the systematic/random errors of tumor reproducibility were 4.5/2.6 (medial-lateral, ML), 5.1/4.8 (cranial-caudal, CC) and 4.0/3.6 mm (anterior-posterior, AP). These errors were significantly decreased to within 3 mm, both post-correction and post-treatment. The corresponding PTV margins were 4.7 (ML), 7.4 (CC) and 5.4 (AP) mm. The changes in the tumor position relative to the vertebral bone displayed systematic/random errors of 2.2/2.0 (ML), 4.1/4.4 (CC) and 3.1/3.3 (AP) mm. The uncertainty of the vertebral bone significantly correlated to the reproducibility of the tumor position (*P* < 0.05), except in the CC direction post-treatment. However, no significant correlation was detected between the relative tumor position, the total treatment time and the tumor reproducibility (*P* > 0.05).

**Conclusions:**

Using ABC for single-breath-hold CBCT guidance is an effective method to reduce the PTV margin of hypofraction radiotherapy for lung cancer. Using ABC, the tumor position was significantly altered relative to the vertebral position. The reproducibility of the tumor position was affected by the vertebral bone but not by the relative tumor position or the total treatment time.

## Background

The precision of radiotherapy, which involves image acquisition, treatment planning and radiation delivery, is affected by respiratory motion [1-3]. Active breathing control (ABC) can suspend breathing during the acquisition of simulation CT or cone beam CT (CBCT), thereby eliminating the artifact of respiratory motion and enabling accurate delineation of the gross tumor volume and image registration. Second, breath-holding during inhalation increases the total lung volume and reduces the lung mass, resulting in a decreased dose to the normal tissue and an increased dose to the tumor [4,5]. Third, eliminating the effects of respiratory motion during dose delivery would increase the precision of dose distribution. The reproducibility of ABC has been validated by fluoroscopy, electronic portal images and repeated CT scans [5-7]. However, the fluoroscopy and electronic portal image methods include inherent uncertainties, as the vertebral bone was used as a surrogate for the tumor position in many cases because of the poor visibility of the tumor [8]. Although the tumor variability was accurately quantified using repeated CT scans [5], performing repeated CT scans outside of the treatment condition is a potential source of uncertainty. CBCT accurately displays the changes in the internal anatomy under the treatment condition. However, the short period of any single breath-hold limits the potential of CBCT. We previously reported the use of single-breath-hold CBCT guidance combined with ABC in liver cancer patients [9]. To our knowledge, there is no other institute that has a successfully used single-breath-hold CBCT guidance. However, it is more challenging to use such a technique for lung tumors due to the associated decline in respiratory function and the heartbeat [10]. Furthermore, tumors in different locations undergo distinct respiratory motion during free breathing (FB), which may influence the tumor reproducibility of the breath-hold method.

In our study, single-breath-hold CBCT images acquired using ABC in a cohort of 83 lung cancer patients (95 tumors) treated with hypofraction radiotherapy were analyzed retrospectively, which may provide the largest cohort reported to date. We investigate the lung tumor reproducibility and the corresponding PTV margin, as well as the relationships between the tumor reproducibility, the relative tumor position, the total treatment time and the uncertainty of the vertebral bone.

## Materials and methods

### ABC

From September 2007 to August 2013, 121 patients who planned to be treated with single-breath-hold CBCT guided hypofraction radiotherapy for lung cancer were trained using modified Active Breathing Coordinator software (version 1.1, Elekta Oncology Systems, Stockholm, Sweden) [9] to follow a specific ABC flowchart. Motion of the tumor or the pulmonary vessels near the tumor were measured via fluoroscopy (Elekta Oncology Systems Synergy^s^, Motion View, version 3.5) for FB and breath-holding using diagnostic computed tomography (CT) as a position reference. During the training session, the patients were placed in the supine position. The airflow through the nose was blocked using a nose clip, medical cotton or a hand. The patient was instructed to inspire deeply before the breath hold, followed by full expiration. Normal breathing was resumed by opening the balloon valve with a hand-held switch or by releasing the mouthpiece. The initial breath threshold was set at approximately 75% (adding 0.1-0.3 L according to the patient’s compliance) of the measured deep inspiration volume (DIV).

**Figure 1:**
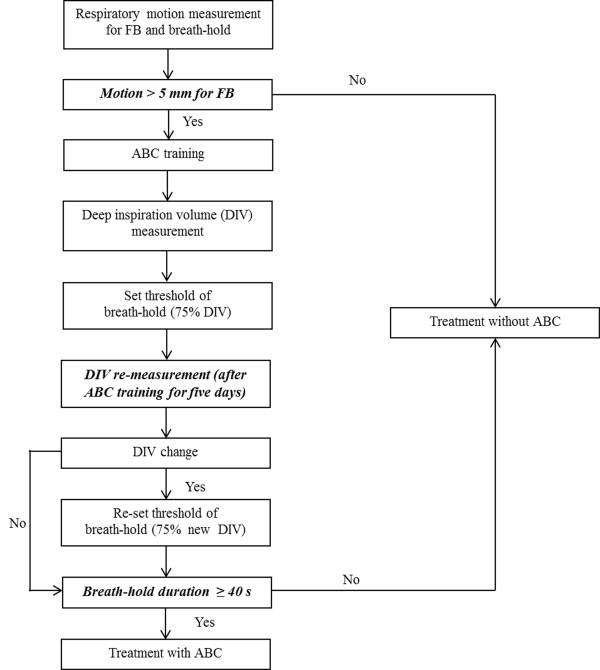
**The modified ABC procedure.** Crucial steps included respiratory motion less than 5 mm, maximum breath-hold duration less than 40 s and deep inspiration volume (DIV) re-measurement after ABC training for five days.

During the initial practice (between 2007 and 2008), four patients complained that the airflow was blocked too early for them to comfortably hold their breath using the initial threshold after three to five training days. Another four patients displayed a significant change in the lung contours between the CBCT and the simulation, leading to a substantial change in the tumor position. For these eight patients, the ABC thresholds were re-set, the CT scans were re-simulated, and the ABC flowchart was amended accordingly. DIV re-measurement after ABC training for five days was added to the flowchart to determine a suitable breath-hold threshold (Figure 1). Patients displaying less than 5 mm of respiratory motion of the tumor or the pulmonary vessels near the tumor [2] were excluded from this study. A planning CT and single-breath-hold CBCT-guided hypofraction radiotherapy plan were performed when the breath-hold duration was greater than 40 s with less than 100 ml threshold fluctuation after ABC training for five days. Four strategies were used to confirm the validity of ABC [9].

### Patients

While following the ABC flowchart, 83 of the 121 lung cancer patients (95 tumors) were treated with single-breath-hold CBCT-guided hypofraction radiotherapy using ABC. Written consent was obtained from all patients prior to enrollment in the study. All cases of primary lesions were confirmed based on biopsies. The study included three patients experiencing Stage I (T1-T2 N0) NSCLC and 80 patients exhibiting lung metastases from lung cancer (n = 42), head and neck cancer (n = 9), breast cancer (n = 6), liver cancer (n = 6), prostate cancer (n = 2), colorectal cancer (n = 5), rectal cancer (n = 4) or sarcoma (n = 6). The diagnosis of metastasis was established via CT, magnetic resonance imaging (MRI) or positron emission tomography-CT (PET-CT). The median gross tumor volume (GTV) was 2.4 cc (range: 0.50-33.0 cc). The median age was 54 years (range 22–78). The Karnofsky performance status (KPS) score was greater than 80 for all patients. The ratio of the forced expiratory volume in 1 s to the forced vital capacity (FEV_1_) was greater than 80%.

### Planning CT and preparation

The patients were placed in the supine position in a stereotactic body frame (SBF, Elekta Oncology Systems) (n = 77) or in blue bags (Elekta Oncology Systems) (n = 6). Planning CTs were performed using a Philips Gemini 16-slice CT with a 2-mm slice thickness from the throat to the liver after administration of an intravascular contrast solution (100 ml of iodine solution (300 mg/ml) administered at 3 ml/s for a volume of 2 ml/kg up to a maximum of 150 ml). According to the literature [11] and our previous results [9], the PTV was generated based on an 8 mm expansion in both the ML and AP directions and a 10 mm expansion in the CC direction around the clinical tumor volume (CTV). The CTV was defined as the GTV + 3 mm. Conformal 3D plans including 6–10 beams were designed using Precise PLAN® (Elekta Oncology Systems) or Pinnacle 9.0 (Philips Medical Systems, Madison, WI). The tumors were subjected to one of seven different doses based on the tumor size and pathology: 26 Gy × 1f (n = 1), 15 Gy × 3f (n = 15), 12 Gy × 4f (n = 37), 10 Gy × 5f (n = 12), 10 Gy × 6f (n = 8), 8 Gy × 7f (n = 16) or 6 Gy × 10f (n = 6).

### CBCT acquisition and registration

In each fraction, all patients underwent pre-correction CBCT imaging using ABC after completing the setup. For some patients, second and third CBCT images were recorded. A second CBCT (post-correction) was acquired to validate the couch shifts. The couch shifts were performed manually with a threshold of 2 mm in any direction. A third CBCT (post-treatment) was acquired to assess the intra-fraction error for some patients according to each patient’s physical state or total treatment time.

**Figure 2:**
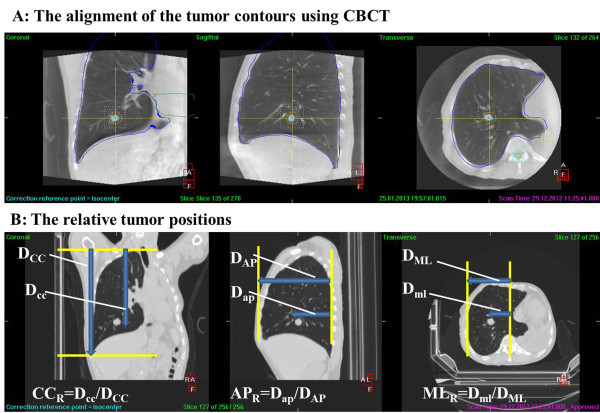
**The alignment of the tumor contours using CBCT and the relative tumor position. A:** The alignment of the tumor contours using CBCT. The tumor contours in the reference CT image were aligned with those of the CBCT image because the tumor was highly visible in the CBCT image. **B:** The relative tumor positions defined in the coronal, sagittal and transverse axes.

Each CBCT imaging was acquired within a single breath-hold, with the breath-hold duration ranging from 40–70 s. Even small tumors are easily visible in all CBCT images (Figure 2A), which facilitates alignment of the reference CT and CBCT images. The tumor reproducibility was evaluated based on the automated alignment of the grey values within the region of interest around the tumor. Subsequently, the tumor contour was manually aligned to verify the automatic alignment results when all rotational errors were set at zero. Note that in some cases, improper matching results were obtained using the grey value alignment, or the images could not be aligned. Therefore, all of the tumor alignment errors were determined using a manual method.

The parameters for CBCT imaging (Elekta Oncology Systems synergys, version 3.5) were 120 kV, 361 frames, 25 mA, 32 mS, bowtie filter, 288.8 mAs, a gantry start angle of 260° or 100°, a gantry stop angle of 100° or 260°, a gantry speed of 3.18°/s and 5.5 frames/s for at least 200 frames.

### Tumor position reproducibility and CTV-PTV margin

The CTV-PTV margin was calculated as the sum of the setup margin (SM) and the internal margin (IM). The amplitude of the tumor motion was defined as IM based on the fluoroscopy measurement data or published results [7,10]. The alignment results were used to calculate the SM using the formula developed by Sonke et al.: SM = 2.5 $\sum +\beta \sqrt{\sigma^{2}+{\sigma_{p}}^{2}}-\beta \sigma_{p}$, where *σ*_*p*_ = 0.64 for the lungs, *β* = 0.84 for SBRT (80% isodose line), Σ represents the systematic error and *σ* represents the random error [12].

### Tumor position changes relative to the vertebral bone

To calculate the changes in the tumor position relative to the vertebral bone, all of the CBCT images were retrieved for retrospective analysis. The vertebral bone uncertainties were evaluated by first automatically aligning the vertebral bones in the reference CT and CBCT images. Subsequently, manual alignments of the vertebral bones were performed if there were incorrect matching results or if the images could not be aligned. For each patient, the difference between the tumor reproducibility and the vertebral bone uncertainties represented the changes in the tumor position relative to the vertebral bone. During data analysis, all rotational errors were neglected, as no 6D treatment could correct for these errors.

### Pearson correlation between the uncertainty of the vertebral bone, the relative tumor position and the reproducibility of the tumor position

The relative tumor positions were defined and measured as previously described by Onimaru [13] (Figure 2B):

CC_R_ = The distance between the lung apex and the caudal portion of the tumor (D_cc_)/The distance between the lung apex and the diaphragm (D_CC_).

ML_R_ = The distance between the body midline and the lateral border of the tumor (D_ml_)/The distance between the body midline and the lateral border of the lung (D_ML_).

AP_R_ = The distance between the posterior portion of the lung and the anterior portion of the tumor (D_ap_)/The distance between the posterior and anterior portions of the lung (D_AP_).

Annotation: CC_R_, ML_R_ and AP_R_ represent the relative tumor positions in the CC, ML and AP directions, respectively.

The correlations between the relative tumor position, the vertebral bone uncertainty and the reproducibility of the tumor position were analyzed using Pearson correlations, and *p* values ≤ 0.05 were considered to be statistically significant. All statistical analyses were performed using SPSS software, version 16.0.1, or Microsoft Excel.

## Results

A total of 1084 CBCT images were obtained from the 83 patients displaying 95 lung tumors, including 474 pre-correction images, 336 second scan images and 238 third scan images.

### Tumor position reproducibility, vertebral bone uncertainty and change in the tumor position relative to the vertebral bone

For the pre-correction scans, the systematic/random errors of the tumor position reproducibility range from 2.6 to 5.1 mm; the corresponding vertebral bone uncertainties were noteworthy, in the range of 3.4-5.5 mm. The reproducibility of the tumor position decreased to within 3 mm in any direction on the second and third CBCT images. However, the reduction in the vertebral bone uncertainty was not comparable to that of the tumor position reproducibility (systematic/random error range from 1.6 to 4.4 mm) of the second and third CBCT images. Similar to the vertebral bone uncertainty, the changes in tumor position relative to vertebral bone displayed systematic/random errors of 2.2/2.0 (ML), 4.1/4.4 (CC) and 3.1/3.3 (AP) mm. The details of these errors are presented in Table 1.

**Table 1:** The tumor position reproducibility, the vertebral bone uncertainties and the change in the tumor position relative to the vertebral bone (mm)

	**The vertebral bone uncertainties**			**The tumor position reproducibility**			**The change in the tumor position**		
	**ML**	**CC**	**AP**	**ML**	**CC**	**AP**	**ML**	**CC**	**AP**
Pre-correction CBCT									
Σ	4.2	5.5	3.4	4.5	5.1	4.0	2.4	4.0	2.7
σ	4.5	5.0	4.9	2.6	4.8	3.6	3.1	4.7	4.1
SM	14.2	18.0	12.6	13.5	16.8	13.0	8.6	13.8	10.3
Second CBCT									
Σ	2.5	3.3	2.7	1.1	1.7	1.1	2.3	3.4	2.5
σ	2.4	3.7	3.2	1.7	2.9	2.2	1.9	4.1	2.8
SM	8.2	11.3	9.4	4.3	6.6	4.6	7.3	12.0	8.5
Third CBCT									
Σ	1.7	4.4	3.2	1.2	2.0	1.4	2.2	4.1	3.1
σ	1.6	3.6	3.4	1.9	2.8	2.7	2.0	4.4	3.3
SM	5.5	14.1	10.9	4.7	7.4	5.7	7.2	13.9	10.4

Notes: Σ: the SD of the individual means; σ: the root-mean-square of the individual SD; SM = set-up PTV margin = 2.5 $\sum +\beta \sqrt{\sigma^{2}+\sigma_{p}^{2}}-\beta \sigma_{p}$, where *σ*_*p*_ = 0.64 for lung, *β* = 0.84 for SBRT (80% isodose line).

### CTV-PTV margin

The calculated SM values are listed in Table 1. The SM values were 5.5, 14.1 and 10.9 mm in the ML, CC and AP directions when evaluated based on the vertebral bone position errors. In contrast, the SM margins were 4.7 (ML), 7.4 (CC) and 5.7 mm (AP) when the tumor position reproducibility was used to analyze the SM.

The tumor motion (IM) along the AP axis was not quantifiable for all patients’ tumors due to the poor visibility in the fluoroscopic images. Only 26 tumors in 24 patients were visible, and the motion amplitude of these tumors was quantified in the CC and ML directions via fluoroscopy. The use of ABC alone decreased the tumor motion to less than 1 mm in both the CC and ML directions for 19 tumors (17 patients). In contrast, the tumor motion amplitudes were 7–21 mm in the CC direction and 1–3 mm in the ML direction for FB. The other 7 tumors (7 patients), which were located near the heart (less than 3 cm), displayed 2–3 mm motion in the CC and ML directions using ABC. For FB, theses amplitudes were 12–17 mm in the CC direction and 3–5 mm in the ML direction.

Compared with FB, there was sharp reduction in the IM in the CC direction upon use of ABC (the IM was detected as 0 mm in the majority of patients). As a result, the CTV-PTV margins were 3.3 (ML), 2.6 (CC) and 2.3 mm (AP) smaller than the clinical margins (8 mm in the ML and AP directions, 10 mm in the CC direction), except for tumors located near the heart (less than 3 cm).

### Pearson correlation between the tumor position reproducibility and the vertebral bone uncertainty

As shown in Table 2, there was a significant Pearson correlation between the tumor position reproducibility and the vertebral bone uncertainty except in the CC direction post-treatment (*P <* 0.05). The Pearson coefficients using the pre-corrected images in the ML, CC and AP directions were 0.795, 0.838 and 0.701, respectively. These Pearson coefficients decreased sharply in all three directions using the second and third CBCT images.

**Table 2:** Pearson correlation between the tumor position reproducibility and the vertebral bone uncertainty

	**Pre-correction**			**Second CBCT**			**Third CBCT**		
	**ML**	**CC**	**AP**	**ML**	**CC**	**AP**	**ML**	**CC**	**AP**
Coefficient	0.80	0.84	0.7	0.34	0.26	0.38	0.31	0.24	0.33
*P*_value_	0.00	0.00	0.00	0.00	0.00	0.00	0.04	0.13	0.04

### Pearson correlation between the relative tumor position, the total treatment time and the tumor position reproducibility

The median relative tumor positions were 0.62, 0.68 and 0.42 in the ML, CC and AP directions, respectively. We did not detect any significant Pearson correlations between the relative tumor position and the tumor position reproducibility in any direction for all CBCT images (*P* > 0.05) (Table 3).

**Table 3:** The relative tumor positions and Pearson correlations between the relative tumor position and SD of the tumor position reproducibility

	**The relative tumor positions**			**Pearson correlation (*P***_**value**_**)**		
	**Max**	**Min**	**Median**	**Pre-correction CBCT**	**Second CBCT**	**Third CBCT**
ML	0.93	0.16	0.62	0.50	0.54	0.49
CC	1.00	0.17	0.68	0.64	0.18	0.36
AP	0.98	0.10	0.42	0.57	0.69	0.19
3D vector	1.50	0.50	1.06	0.90	0.22	0.07

The maximum, minimum and median total treatment time (from the pre-correction to the post-treatment CBCT images) was 49, 9 and 20 min, respectively. There was no significant Pearson correlation between the total treatment time and the tumor position reproducibility (post-treatment) in any direction (*P* > 0.05).

## Discussion

While following specific ABC training program using an appropriate threshold, 69% of the lung cancer patients successful used the ABC method. It should be noted that this percentage would be sharply decreased when considering patients exhibiting less than 80% FEV_1_. We strongly recommend DIV re-measurement after ABC training for five days to determine a suitable breath-hold threshold. In the current study, we found no correlation between the maximum breath-hold time and the breath-hold threshold, suggesting that a patient exhibiting a low breath-hold threshold may exhibit long breath-hold duration.

Consistent with previous reports [5,6], the inter-fraction tumor position reproducibility using ABC displayed substantial systematic and random errors, particularly in the CC direction, suggesting that image guidance is essential. Under conventional fraction treatment, the inter-fraction variability of the tumor position was substantial due to tumor regression and pathological changes over the course of the treatment [5,14-16]. In our study cohort, the tumor was not likely to undergo substantial regression due to the brief treatment duration (maximum two weeks). Meanwhile, no pathological changes occurred over the course of treatment. From our results, the vertebral bone uncertainties were larger than those reported by Brock J [5] (3.4-5.5 mm range versus 1.4-3.7 mm range). Moreover, there was a significant correlation between the tumor position reproducibility and the vertebral bone uncertainty. According to these results, we conclude that setup error was a fundamental factor affecting the inter-fraction tumor position reproducibility [17].

The systematic and random errors of the tumor position were significantly decreased to within 3 mm both post-correction and post-treatment, clearly indicating the benefit of image guidance. As the corresponding vertebral bone uncertainties were larger, these errors were corrected according to the tumor position rather than the vertebral uncertainties. The small Pearson Correlation coefficients revealed that the vertebral bone uncertainties exerted little contribution to the post-correction and post-treatment tumor positions. Previous reports have indicated little change in the lung volume between different breath-holds [5,14,15], which can induce errors in the tumor position. Tumors in the upper lobes tend to display less motion than tumors in the lower lobes, making them less sensitive to changes in the lung volume. In our study cohort, the tumor positions were in the lower and lateral portions of the lung because the median relative tumor positions were greater than 0.5 in the ML and CC directions, making these tumors more sensitive to changes in the lung volume. Furthermore, Shah C [18] reported that different immobilization devices can influence the post-correction residual error. In our study, no significant differences were detected between the immobilization devices used, which may be due to the small number of samples in the blue bag group (n = 6) and the fact that both of these devices are vacuum bags. Furthermore, no significant associations were detected between the tumor position reproducibility and the total treatment time, which was consistent with the report of Peguret [19]. Fortunately, as a result of the integration of different uncertain causes, the tumor position reproducibility under single-breath-hold CBCT guidance using ABC was at a clinically acceptable level.

The limitations of our study include the inability to investigate the lung volume due to the small (S20) FOV for the CBCT scan and that the registration results of the second and third CBCT images do not represent the actual intra-fraction error. To perform a simple calculation of the CTV-PTV margin, the post-treatment tumor position reproducibility was used to determine the SM, and the tumor motion measured via fluoroscopy was used to determine the IM. Using ABC, the IM was significantly reduced in the CC direction (7–21 vs. less than1 mm). However, ABC displayed little ability to reduce the motion amplitude in the ML direction (1–5 vs. 1–3 mm). Seppenwoolde Y reported a 1–4 mm range of tumor motion (most prominent in the lateral direction) caused by the heart beat [10], and only 0–2 mm motion was detected using ABC [7]. Considering these results, we conclude that when using ABC, consideration of the IM was unnecessary for tumors far away from the heart, but the tumor motion should be measured to determine the IM of tumors located near the heart (less than3 cm), especially for those near the left ventricle. Our results revealed that single-breath-hold CBCT guidance using ABC is effective for the management of respiratory motion because the CTV-PTV margin was significantly smaller than that reported by Brock (11.9 (ML), 18.1 (CC) and 11.9 (AP) mm) [5] and Panakis (8.3 (ML), 12.0 (CC) and 9.8 (AP) mm) [7].

The results of changes in the tumor position were obtained between the CBCT and planning CT scans, representing the inter-fraction error. Weiss [14] reported that changes in the tumor position relative to the vertebral bone displayed inter-fraction systematic and random errors of 3.0-4.6 mm and 2.6-2.9 mm, respectively. These errors were significant and in agreement with our findings. In our opinion, the changes in the tumor position primarily contributed to the changes in the tumor baseline position from the CT simulation to the treatment time. The changes in the tumor baseline position can be explained by the changes in the breath-hold lung volume post-CT simulation. Changes in the lung volume are primarily affected by two causes: (1) due to differences in the flow rate between individual patients, the delay between the activation of the ABC when the threshold volume is reached and the closure of the balloon valve causes a variation in the lung volume [6]; (2) differences in the residual volume prior to inspiration when performing the breath-hold. These two causes could have significant effects in most patients after ABC training [15]. However, the lung volume could not be analyzed in the current study. The present data exclusively displayed large PTV margins (range 7.2-13.9 mm) when using the vertebral bone position as a surrogate for the tumor position. In other words, greater care should be taken when using 2-D image guidance (portal images or fluoroscopy) to perform lung tumor radiotherapy using ABC.

The location of the lesion affects the magnitude of tumor motion during FB [13]. However, Peng [4] noted that the extent of tumor motion during normal respiration does not influence the tumor position under breath-hold conditions. In our study, we also did not detect any correlation between the relative tumor position and the tumor position error. These results suggest that tumors at lower locations or displaying larger excursion during FB would be better candidates for the use of ABC.

Purdie TG [20] reported an intra-fraction error shift when the interval between the initial localization and the repeated CBCT was more than 34 min. In our study, total treatment time was less than 34 min in 96% of the cases. Furthermore, post-correction error, post-treatment error and post-treatment error minus post-correction error only represent the errors during this limited period (40–70 seconds during CBCT imaging), which were determined based on the changes in the lung volume between different breath-holds. Therefore, the reproducibility of the tumor position was not affected by the total treatment time in our study.

## Conclusions

By following a specific ABC flowchart, single-breath-hold CBCT guidance can be performed successfully in 69% of lung cancer patients. Using ABC for single-breath-hold cone beam CT guidance is an effective method to reduce the PTV margin of hypofraction radiotherapy for lung tumors. Using ABC, the tumor position reproducibility was affected by the vertebral bone but not by the relative tumor position or the treatment time. The lung tumor position changed significantly relative to the vertebral position, which should be studied further. Large PTV margins are recommended when using the vertebral bone as a surrogate for the tumor position in lung cancer patients.

## Competing interests

The authors declare that they have no competing interest.

## Authors' contributions

RZ designed the study and drafted the manuscript. JW revised the manuscript. FX and NC analyzed the data. LZ, LL, JZ and XJ helped to collect the data. YL and SB contributed equally to the conception of this study and the final approval of the version to be published. All authors read and approved the final manuscript.
